# Implementing an operative step-based typology for improved neurosurgical training assessment

**DOI:** 10.1016/j.bas.2023.101783

**Published:** 2023-07-11

**Authors:** Aaron Lawson McLean

**Affiliations:** Department of Neurosurgery, Jena University Hospital – Friedrich Schiller University Jena, Jena, Germany

**Keywords:** Assessment, Educational, Curriculum, Neurosurgery, Professional competence, Surgical procedures, Operative, Teaching methods

To the Editor,

## Authorship statement

ALM was responsible for all aspects of the work, including drafting and revising the manuscript critically for important intellectual content, and final approval of the version to be published.

## Conflict of interest statement

The author declares no potential conflicts of interest with respect to the research, authorship, and/or publication of this article.

## Funding statement

No external funding was secured for this study.

## Patient consent for publication

Not applicable.

## Ethics approval

Not applicable.

The report by Whitfield et al. on the “European training requirements in neurological surgery: A new outcomes-based 3 stage UEMS curriculum” has invigorated the discourse around the efficacy and relevance of neurosurgical training practices (Whitfield et al., 2023). A paradigm shift towards outcomes-based models from traditional time-based ones necessitates a meticulous and comprehensive record of operative experiences, an aspect that existing systems might inadequately portray due to their holistic operation-based approach.

This conventional approach to surgical education risks misrepresenting trainees' actual operative involvement, constraining the identification of explicit learning needs. A resolution to this issue can emerge from an enhanced approach that deconstructs operations into discrete, manageable components, promoting detailed experience logging. Two examples of such a step-based typology are shown in Table 1.

**Table 1:** Deconstruction of two contrasting neurosurgical operations: a complex cranial procedure (microvascular decompression for trigeminal neuralgia) and a relatively simpler procedure (ventriculoperitoneal shunt placement). The procedures have been broken down into constituent steps that mirror the progression of a trainee's skill acquisition in line with the UEMS curriculum, offering an improved system for recording and evaluating operative experience.

	Microvascular Decompression (MVD) for Trigeminal Neuralgia	Ventriculoperitoneal (VP) Shunt Placement
Step 1	Position patient and prepare surgical field	Position patient and prepare surgical field
Step 2	Perform skin incision and create a bone flap	Perform scalp incision
Step 3	Open dura and expose cerebellar hemisphere	Drill burrhole and insert ventricular catheter
Step 4	Identify and expose trigeminal nerve root entry zone	Connect ventricular catheter to valve
Step 5	Place Teflon felt between nerve and offending vessel	Tunnel catheter under skin from head to abdomen
Step 6	Verify adequate decompression	Insert distal end of catheter into peritoneal cavity
Step 7	Close dura, replace bone flap and close skin incision	Connect distal catheter to valve, check valve function, and close skin incision
Step 8	Post-operative assessment and planning for next steps	Post-operative assessment and planning for next steps

A granular look at surgical procedures, especially in neurosurgery with its complexity and sophistication, enables more precise documentation of the trainee's participation in multifaceted operations like tumour resections or complex spinal cases. This operation deconstruction aligns seamlessly with the UEMS curriculum's stage-wise progression, allowing trainers to accurately gauge trainees' proficiency at each stage and adjust training plans accordingly.

Moreover, the incorporation of this deconstructed approach within pre and post-operative briefings facilitates a culture shift towards proactive planning and inclusive education. Detailed discussions about trainee involvement in individual components of an operation could contribute to reducing the disparity between training needs and delivery.

The value of this innovative approach becomes particularly salient during disruptions, like the recent global health crises, where maximizing each training opportunity is crucial. By adopting a detailed and proactive methodology, we can bolster the resilience of neurosurgical training.

Nevertheless, this proposal does not come without its challenges. While the deconstructed logbook concept applies well to procedures with defined steps, it may be less effective for unpredictable operations. Also, the additional administrative tasks for trainees must be carefully balanced against the potential benefits. However, the potential to accurately portray experience, identify training needs, and cultivate a culture of continuous learning should spur further exploration of this approach.

In concert with the outcomes-based UEMS curriculum, the proposed deconstruction of operations and associated changes could transform neurosurgical training. By facilitating a more accurate representation of trainee experience and a better reflection of stage-wise progression, we move towards producing neurosurgeons who are not just competent but are also confident practitioners.

These innovations represent a significant stride towards training that is not just responsive but also predictive, aligning seamlessly with the objectives of the UEMS curriculum, and ready to meet the dynamic demands of neurosurgical practice.

Sincerely,

Aaron Lawson McLean.

## Declaration of competing interest

The author declares that they have no known competing financial interests or personal relationships that could have appeared to influence the work reported in this paper.
